# Impact of replacing fat with pearl millet fibers on the bioactivity and quality of beef burger

**DOI:** 10.1038/s41598-025-30464-w

**Published:** 2025-12-15

**Authors:** Mohamed E. Salem, Samar M. Aref, Mohamed Y. Abouelwafa, Safaa M. Mokbel, Alzahraa M. Motawei, Ammar AL-Farga, Tamer E. Moussa-Ayoub

**Affiliations:** 1 Food Technology Department, Faculty of Agriculture, Suez Canal University, Ismailia, 41522 Egypt; 2 Dairy Department, Faculty of Agriculture, Suez Canal University, Ismailia, 41522 Egypt; 3 Food Industries Dept., Faculty of Agriculture, Mansoura University, Mansoura, 35516 Egypt; 4 State Key Laboratory of Food Science and Technology and School of Food Science and Technology, Jiangnan University, 1800 Lihu Avenue, Wuxi, 214122 People’s Republic of China

**Keywords:** Pearl millet bran, Low-fat beef burger, Microbial quality, Cooking characteristics, Sensory attributes, Antimicrobials, Bacteria, Diseases

## Abstract

This study focused on developing a low-fat beef burger by replacing added beef fat with pearl millet bran. The control sample (T1) consisted of 85% lean meat and 15% added animal fat. Pearl millet bran (PMB) was incorporated at varying ratios: 0% (T1), 5% (T2), 10% (T3), and 15% (T4) of the total content in the beef burgers, in place of the added beef fat. Analyses using the Folin-Ciocalteu and DPPH assays showed that the addition of pearl millet bran increased both phenolic content and antioxidant activity in the burgers. Quality indices, particularly peroxide and TBARS values after 3 days of storage, were lower for T4 (1.77 meq O_2_/kg fat and 0.38 mg MDA/kg fat) compared to T1 (3.12 meq O_2_/kg fat and 0.78 mg MDA/kg fat). Additionally, PMB improved the cooking characteristics of the resultant burgers. The cooking loss decreased from 36.36% in T1 to 15.20% in T4, while the shrinkage ratio reduced from 31.10% in T1 to 13.29% in T4. The firmness of the burger samples improved with the addition of pearl millet bran. The pearl millet bran increased all color parameters. However, this did not impact negatively on the acceptance of cooked beef burgers. In terms of sensory attributes, sample T2 was significantly preferred over T1, T3, and T4. Overall, this study highlights the potential of pearl millet bran as a natural bioactive fat replacer for creating a healthier processed meat product with desirable eating characteristics.

## Introduction

Dietary fiber plays a crucial role in controlling blood sugar, excreting cholesterol, and regulating the gastrointestinal tract^1^. Food products typically low in dietary fiber and high in saturated fat and cholesterol place consumers at risk for coronary heart disease (CHD), obesity, diabetes, cancer, and other cardiovascular diseases^2^.

In 2022, the fast-food market achieved a major milestone with sales reaching $731.65 billion^3^. This market is expected to continue expanding due to changing consumer tastes and lifestyles, rapid urbanization, and a growing demand for quick and convenient food options. Burgers are commonly associated with fast food restaurants because they are convenient, affordable, and widely available. Meat and chicken products such as burgers, patties and sausages are widely consumed food products due to their appreciable sensory properties, nutritional quality, and low coat^4^. But consumers’ concern about the quality of processed meat products have increased, leading to advancements in healthier reformulations aimed at improving nutritional quality by reducing saturated fat content^5,6^. A potential benefit of incorporating dietary fibers into diets is enhancing consumer’s health and improving functional characteristics that alter the sensory aspects of the final product. One of the key functional attributes is their capacity to act as a fat substitute^7^.

Pearl millet (*Pennisetum glaucum*) is a drought-resistant plant, and its grain is nutrient-dense and gluten-free, making it an excellent choice for various food applications such as sweets, bread and cupcakes^8,9^. Globally, pearl millet is recognized as the eighth most productive crop, following wheat, rice, barley, corn, rye, oats, and sorghum^10^. Pearl millet contains fibers, minerals, proteins, and antioxidants in comparable or even greater amounts than some traditional grains like rice and maize^11^. 100 g of pearl millet provides about 378 cal, and contains 8.0% water, 67.0% carbohydrates, 11.0% protein, 3.0% lipids, 3.0% ash, and 7.0% dietary fibers^12^. Pearl millet contains different minerals, including calcium, magnesium, phosphorus, sodium, potassium, zinc, copper, and iron^1^. The bran layer of pearl millet is a good source of B-complex vitamins and antioxidants such as flavonoids and phenolic acids, making it suitable for exploiting in large-scale production of foods^8,13,14^. Pearl millet grains contain significant amounts of cinnamic acid derivatives, such as hydroxycinnamic, coumaric, ferulic, and sinapic acids, while they have lower levels of benzoic acid derivatives, including hydroxybenzoic acid, gallic acid, p-hydroxybenzoic acid, vanillic acid, syringic acid, and protocatechuic acid^11^. Nani et al.^15^ reported that pearl millet contains syringic acid (7.4 μg/g), ferulic acid (199 μg/g), gallic acid (15.3 μg/g), and p-coumaric acid (1350 μg/g). It is preferred to consume the pearl millet in its whole grain or bran form, as phenolic compounds are generally not evenly distributed throughout the grain but are primarily accumulated in the pericarp^8^. Pearl millets exhibit antioxidant, anti-inflammatory, enzyme inhibition and antibacterial potentials^16^.

Following microbial deterioration, oxidative processes, primarily linked to lipids, rank as the second leading cause of spoilage in meat products^16^. Therefore, when formulating meat products, it is advisable to include a natural ingredient that possesses both antioxidant and antimicrobial properties. However, developing low-fat meat products presents a challenge because reducing fat content can lead to changes in texture and taste, which may lower product acceptability. As a result, the meat industry is exploring new dietary ingredients that can replace fats without raising production costs, while also maintaining the sensory qualities that consumers expect. There have been several trials exploring the benefits of using pearl millet and in various meat products, such as chicken nuggets and chicken sausages. However, to the best of our knowledge, there is a lack of information on the use of pearl millet bran (PMB) in beef burgers. In this context, the objective of this study was to evaluate the effects of replacing fat levels and the addition of pearl millet bran (PMB) dietary fiber on some physical, chemical, microbiological, and sensory characteristics of beef burger.

## Material and methods

Fresh 15 kg of beef striploin muscle (*Longissimus lumbroum*) containing beef backfat were sourced randomly from a local market located in Ismailia, Egypt. The experiment was designed and carried out in triplicates, and samples of each replicate made from 5 kg of meat. Spices, including black pepper, nutmeg, clove, onions, and fresh garlic, were purchased from a local market. Pearl millet (*Pennisetum americanum L*., cv. Shandawel-1) was procured from Crop Research Institute, Agricultural Research Center, Giza, Egypt. Additionally, other analytical-grade chemicals were acquired from Sigma-Aldrich Co., St. Louis, MO, USA and microbial media were from Merck, Darmstadt, Germany.

### Preparing millet flour to separate the bran

The millet grains were sieved to remove any debris or broken grains. The grains were ground into granules using a laboratory mill (Brabender Automat Mill Quandrumat Senior, Germany) and sieved using a 150 μm screen^9^. The millet bran was then obtained and placed in high-density polyethylene bags and stored in a freezer at − 18 °C until use.

### Preparation of beef burgers

Fresh beef striploin and beef fat were minced separately using SAP Meat Mincer TC22 (Italy) with a 4 mm steel plate. The beef burgers were prepared by blending the minced meat (85 g), sodium chloride (1.5 g), seasoning (0.022 g) comprising cloves, black pepper, and nutmeg), and sodium tripolyphosphate (0.03 g) in a Kenwood meat mixer (Kenwood Ltd., Havant, UK) for 5 min. The meat mixture was divided into four equal parts which were then mixed with beef backfat and pearl millet bran to produce the experimental treatments (Table 1). Burgers weighing 50 ± 1 g were formed using a hand-press maker (Italman, Italy) with a diameter of 9 cm. The burger treatments were stored at 4 ± 1 °C for 3 days and analyzed on days 0, 3 of the storage periods. Samples were selected at random for analysis every two days during this time.

**Table 1:** Weights (g) of beef burger ingredients.

Treatment	Lean meat	Beef backfat	Peearl millet bran	Sodium chloride	Seasoning	Sodium tripolyphosphate
T1	85	15	–	1.5	0.022	0.03
T2	85	10	5	1.5	0.022	0.03
T3	85	5	10	1.5	0.022	0.03
T4	85	–	15	1.5	0.022	0.03

### Proximate composition analysis

The moisture content, crude protein, crude fat, crude fiber and ash contents were determined in Pearl millet bran as described by AOAC^17^.

### Total phenolic compounds content determination

A sample of 1 g of uncooked beef burger was extracted using 25 mL of 80% methanol. The mixture was shaken for two hours and then centrifuged for 20 min at 3000 rpm. The total phenolic compounds content (TPC) in the methanolic extracts was determined using the Folin-Ciocalteu method as described by Badawy et al*.*^18^ with slight modifications. A 100 μL aliquot of the methanolic extract was mixed with 900 μL of a tenfold diluted Folin-Ciocalteu phenol reagent (1:10 diluted with purified water) and allowed to sit for 5 min at room temperature. Following this, 0.75 μL of a 7% sodium bicarbonate solution was added, and the mixture was vortexed for 30 s before being left to stand for 90 min at room temperature. The absorbance was measured at 725 nm using a spectrophotometer (PG Instruments Ltd., Felsted, Dunmow, UK). A calibration curve using gallic acid (ranging from 0 to 1.00 mg/mL) was created under similar conditions. All values were expressed as the mean (mg of gallic acid equivalents/g of dry weight of three replicates.

### Antioxidant activity determination

The DPPH method was employed to assess the antioxidant activity of the pearl millet bran and uncooked beef burgers sample as described by Aref et al.^19^, with some modifications. To create the stock reagent solution, 22 mg of DPPH was dissolved in 50 mL of methanol (1 × 10^3^ M) and stored at − 20 °C until needed. A working solution (6 × 10^5^ M) was prepared by mixing 6 mL of the stock solution with 100 mL of methanol to achieve an absorbance of 0.8 ± 0.02 at 515 nm. For the assay, 0.1 mL of the prepared methanolic extract was combined with 3.9 mL of the DPPH solution, and the mixture was allowed to react for 30 min. The absorbance (A) was then measured at 515 nm, with the extraction solvent used as a control. The scavenging activity was calculated using the following formula:

$${\text{Scavenging}}\,{\text{activity}}\,\left( \% \right) = \left[ {\left( {{\text{A control}}{-}{\text{A sample}}} \right)/{\text{A}}\,{\text{control}}} \right] \times {1}00$$

### Thiobarbituric acid reactive substances (TBARS)

Lipid oxidation of the uncooked beef burger was determined using the distillation of 2-thiobarbituric acid (TBA) procedure of Halvorsen and Kvernenes^20^ with slight modification. Five mL distillate of 10 g meat sample was reacted with TBA reagent (0.02 M) in boiling water bath for 35 min. A blank was prepared with 5 mL of distilled water and 5 mL TBA reagent. After cooling, the absorbance of the resultant pink color was measured at 532 nm using a spectrophotometer (PG Instruments Ltd., Felsted, Dunmow, UK). TBA values (mg MDA/kg of sample) were calculated by multiplying the absorbance by the factors 7.8.

### Peroxide value

The method of Badawy et al.^18^ was used to determine the peroxide values of uncooked beef burger samples as follows: 150 g of sample was homogenized for 3 min with 250 mL of chloroform in a blender (Matsushita ELEC. IND. Co. Ltd., Japan), and then the mixture was filtered. 37 mL of glacial acetic acid and 1 mL of freshly prepared saturated potassium iodide solution were added to 25 ml of the obtained filtrate. After allowing the solution to stand with occasional swirling for exactly 1 min, a 30 ml of water was added and titrated with 0.01 N sodium thiosulphate.

Peroxide value (PV) was calculated using the following equation as meq O_2_/Kg sample:

$$\begin{aligned} {\text{PV}} = & \,\left[ {{\text{Titration}}{\mkern 1mu} \left( {{\text{ml}}} \right) \times {\text{N}} \times 1000} \right]/{\text{Weight}}{\mkern 1mu} \left( {\text{g}} \right){\mkern 1mu} {\text{of}}{\mkern 1mu}\, {\text{sample}}{\mkern 1mu} \,{\text{in}}{\mkern 1mu}\, {\text{the}}{\mkern 1mu} \,{\text{filtrate}} \\ {\text{N = }} & \,{\text{ Normality \,of \,sodium \,thiosulphate}}{\text{.}} \\ \end{aligned}$$

### Microbiological analysis of uncooked beef burgers

To conduct microbiological analysis, ten grams of each mixed sample were aseptically collected, and 90 ml of sterile buffered peptone water was added. The samples were then homogenized for two minutes. The aerobic plate count (APC) and the psychotropic counts were determined using the pour plate method. Serial dilutions were made, and 1 ml of each dilution was added to plate count agar media (Merck, Darmstadt, Germany). The plates were incubated for 2 days at 35 °C for APC and 10 days at 7 °C for TPC, respectively^19^. To detect yeast and molds, plates containing 100 µg/ml of Chloramphenicol were used and incubated for 48 h at 25 °C. For lactic acid bacteria, MRS agar was utilized as the growth medium, with plates incubated for 2 days at 35 °C. The total coliform count was determined using violet-red bile (VRB) agar as the medium, with incubation at 35 °C for 18–24 h. The results were presented as log CFU per gram of sample.

### Color measurements of beef burgers

The color parameters of uncooked beef burger samples were measured with a Minolta color reader CR-10 (Minolta Co. Ltd., Osaka, Japan) for the CIE color values: L^*^ (lightness), a^*^ (redness), and b^*^ (yellowness).

#### Cooking characteristics of beef burgers

Five burgers from each formulation were cooked for two minutes at approximately 180 °C on a preheated electric grill (WA-BBQ 01, White Whale, China). After turning them over, they were cooked for another two minutes until the core temperature reached 80 °C. The burgers were then cooled for one hour at 21 °C before being weighed. Three burgers from each batch were weighed, and their thickness and diameter were measured both before and after cooking at room temperature. Shrinkage (%) and cooking loss (%) were calculated using the following equations:

$$\begin{aligned} \% \,{\text{Shrinkage}} = & \,{\text{Raw}}\,{\text{diameter}} - {\text{Cooked}}\,{\text{diameter/Raw}}\,{\text{diameter}} \times {1}00 \\ \% \,{\text{Cooking}}\,{\text{loss}} = & \,{\text{Cooked}}\,{\text{weight/raw}}\,{\text{weight}} \times {1}00 \\ \end{aligned}$$

#### Sensory analysis

Burgers were cooked using an electric grill (WA-BBQ 01, White Whale, China) at approximately 180 °C for 2 min, then flipped and cooked for an additional 2 min. The cooked burgers were given separately to 30 semi-trained panelists from the Department of Food Technology, University of Suez Canal, Ismailia, Egypt. Grilled samples were served warm to panelists with randomly coded numbers. Panel members were asked to evaluate the samples for color, odor, taste, tenderness, juiciness, and overall acceptability using a 7-point hedonic scale (1 = dislike highly, 2 = dislike very much, 3 = dislike slightly, 4 = neither like nor dislike, 5 = like slightly, 6 = like very much, and 7 = like highly).

#### Statistical analysis

All measurements were conducted in triplicate except color measurement and sensory evaluation, and data are presented as mean ± SD. Two-way completely randomized analysis of variance (ANOVA) accompanied with Duncan’s multiple range test was used to estimate the significance at (*p* < 0.05) level between the investigated treatments using SPSS software (version 17.0 for Windows, SPSS Inc., Chicago, USA).

## Results and discussion

### Proximate composition of pearl millet bran (PMB).

The results indicated that pearl millet bran (PMB) contains 4.5% moisture, 1.21% crude protein, 0.57% crude fat, 35.9% crude fiber, and 1.20% ash. A similar composition was reported by^8,21^. Numerous studies have demonstrated that PMBs are rich in phenolic compounds, natural antioxidants, fiber, and other essential components needed for both children’s and adults’ healthy growth.

### Total phenolic content and antioxidant activity of uncooked beef burger

**Fig. 1:**
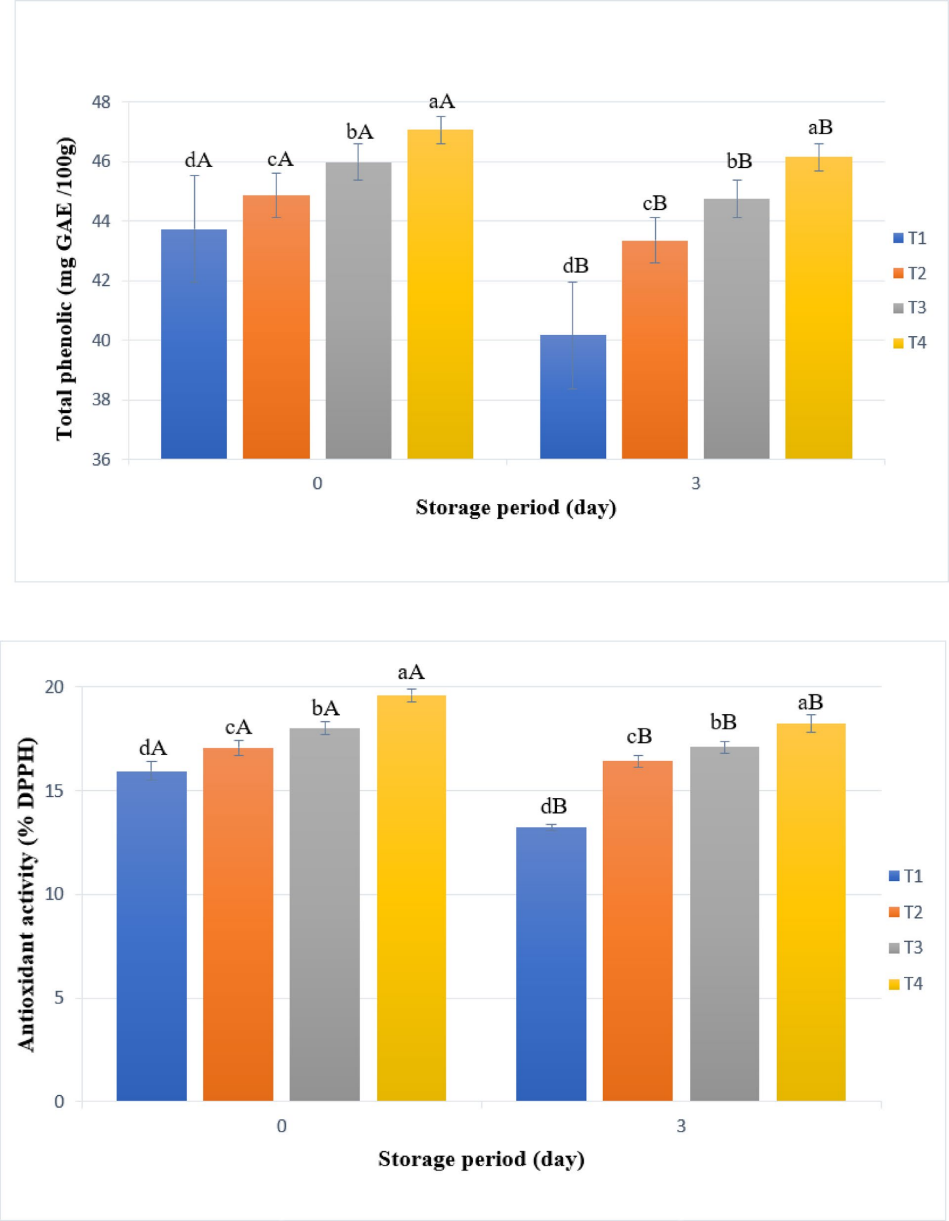
Total phenolic compounds and antioxidant activity of uncooked beef burger samples formulated with different concentrations of pearl millet bran during storage at 4 °C. T1: control, T2: pearl millet bran5%, T3: pearl millet bran10%, T4: pearl millet bran15%. Each value represents the mean of three replicates ± SD. Means that have different lowercase letters (treatments) and uppercase letters (storage time) indicate significant differences at a significance level of *p* < 0.05.

Figure 1 shows that the phenolic content of beef burgers increased with the addition of millet bran. Specifically, the total phenolic content, as measured by the Folin-Ciocalteu assay, rose from approximately 43.74 mg GAE/100 g in T1 to about 47.07 mg GAE/100 g in T4. The phenolics are primarily accumulated in the pericarp and are not evenly distributed throughout the endosperm^8,22^. While during storage the phenolic content of all samples decreased over time may be to the oxidation, T4 experienced the least decline at just 1.95%. In contrast, T4 showed the greatest overall reduction in phenolic content, totaling 8.18% (Fig. 1).

The results regarding the antioxidant activity of the produced beef burgers, as shown in Fig. 1, indicate that an increasing ratio of millet bran led to enhanced antioxidant activity. Specifically, the addition of more millet bran correlated with a greater reduction in DPPH radicals regarding to the increase in total phenolics. In T1, the reduction in DPPH radicals was 15.95%, while in T4, it reached 19.61% after 30 min of reaction. This increase in antioxidant activity is likely due to the presence of phenolic compounds, as there is a positive correlation between phenolic content and antioxidant activity in foods^22^. Different studies have demonstrated that whole grain millet and its bran are rich in phenolic compounds, including flavonoids and phenolic acids, making them a natural source of antioxidants^8^. However, it is important to note that the antioxidant activity of all produced beef burgers decreased over the storage period may be due to oxidation of phenolics and other active compounds (Fig. 1).

### Peroxide, and TBARS values of beef burgers

Peroxide value (PV) is used to assess the quality of oils and fats with regards to lipid oxidation. According to Behbahani et al.^23^, meat can have a maximum PV limit of 7.0 meq O_2_/kg of fat. In the present study, all samples exhibited PV lower than 7.0 meq O_2_/kg of fat (Table 2). Antioxidants can slow down the process of lipid oxidation, which may occur in oils, fats, and food items containing^24^. While the peroxide value of the control beef burger doubled after being stored for 3 days, the PV in beef burgers containing 15% pearl millet bran (T4) increased by only about 11% during the same storage period. These findings suggest that increasing the percentage of fat replaced with pearl millet bran can help slow down lipid oxidation in beef burgers. Baioumy et al.^23^ reported that using a plant-based matrix in place of animal fat improved both the quality and shelf life of beef burger samples. However, the phenolic compounds in millet bran may play a significant role in preventing lipid oxidation in these burgers.

**Table 2:** Changes in peroxide value (meq O_2_/kg fat) and TBARS (mg MDA/kg) of uncooked beef burger samples formulated with different concentrations of pearl millet bran during storage at 4 °C.

Treatments	Storage period (day)		Mean
	0	3	
Peroxide value (meq O_2_/kg fat)			
T1	1.64 ± 0.01	3.12 ± 0.09	2.38^a^
T2	1.62 ± 0.02	2.63 ± 0.03	2.12^b^
T3	1.59 ± 0.02	2.02 ± 0.04	1.80^c^
T4	1.57 ± 0.05	1.77 ± 0.08	1.67^d^
Mean	1.60^B^	2.38^A^	
TBARS (mg MDA/kg fat)			
T1	0.39 ± 0.01	0.78 ± 0.09	0.59^a^
T2	0.35 ± 0.0	0.45 ± 0.02	0.4^b^
T3	0.31 ± 0.01	0.41 ± 0.04	0.36^bc^
T4	0.28 ± 0.05	0.38 ± 0.07	0.33^c^
Mean	0.33^B^	0.50^A^	

T1: control, T2: pearl millet bran5%, T3: pearl millet bran10%, T4: pearl millet bran15%

Each value represents the mean of three replicates ± SD.

Means that have different lowercase letters (treatments) and uppercase letters (storage time) indicate significant differences at a significance level of *p* < 0.05.

The values of thiobarbituric acid reactive substances (TBARS) are also shown in Table 2. The TBARS value of control beef burgers increased from 0.39 mg MDA/kg to 0.78 mg MDA/kg after 3 days of cold storage. In contrast, the addition of pearl millet bran reduced the increase in TBARS across all enriched beef burgers compared to the control (Table 2). For instance, the TBARS value in sample T4 increased by only 26% after 3 days of storage, from 0.28 to 0.38 mg MDA/kg fat. However, the TBARS values in millet bran-enriched beef burgers have increased after three days of cold storage (Table 2). Aly et al.^12^ noted that adding more pearl millet flour to the beef burger reduced the TBA value. Furthermore, Behbahani et al.^23^ noted that meat and meat products can have up to 1 mg of MDA per kilogram of fat. In this present study, all samples including the control sample exhibited TBARS values lower than 1 mg of MDA per kilogram of fat even after three days of cold storage (Table 2).

### Impact of pearl millet bran on microbial load in beef burger

This study evaluated the antimicrobial effects of pearl millet bran (PMB) on the total aerobic plate count (APC), lactic acid bacteria (LAB), psychrophilic bacteria, and coliform bacteria in uncooked beef burgers. Initially, the APC of the control sample was approximately 4.54 logs CFU/g, which increased to about 6.37 logs CFU/g after three days of cold storage at 4 °C (Table 3). According to the International Commission on Microbiological Specifications for Foods^25^, the maximum permitted APC level for fresh beef is 7 logs CFU/g. The results presented in Table 3 indicate that the addition of pearl millet bran significantly reduced the APC, psychrophilic bacteria, and coliform bacteria at the beginning of the storage period. Over the duration of storage, the mean counts of all microbial groups decreased as the amount of pearl millet bran increased. Additionally, both LAB and psychrophilic bacteria counts gradually declined with the addition of pearl millet bran (Table 3). as noticed, there was a slight impact of pearl millet bran on reduction of microbial load. This aligns with previous studies, which reported that pearl millet has antimicrobial properties against various pathogens and can help prevent aflatoxin poisoning. Also noted that the total number of bacteria decreased slightly as the amount of pearl millet powder added increased^26^.

**Table 3:** Changes in aerobic plate count (APC), lactic acid bacteria (LAB), pychrophilic bacteria, and coliform bacteria (log CFU/g) of uncooked beef burger samples formulated with different concentrations of pearl millet bran during storage at 4 °C.

Treatments	Storage period (day)		Mean
	0	3	
Aerobic plate count (APC)			
T1	4.54	6.37	5.45^a^
T2	4.64	5.34	4.99^b^
T3	4.36	6.48	5.42^a^
T4	3.83	5.35	4.59^c^
Mean	4.34^B^	5.88^A^	
Lactic acid bacteria (LAB)			
T1	4.77	6.92	5.84^a^
T2	4.68	6.87	5.78^a^
T3	3.95	7.96	5.96^a^
T4	3.34	6.14	4.74^b^
Mean	4.18^B^	6.97^A^	
Psychrophilic bacteria			
T1	4.07	5.65	4.86^a^
T2	3.43	5.23	4.33^b^
T3	3.30	4.67	3.98^c^
T4	2.60	3.98	3.29^d^
Mean	3.35^B^	4.88^A^	
Coliform bacteria			
T1	3.23	ND	1.61^b^
T2	3.53	ND	1.76^a^
T3	3.00	ND	1.5^b^
T4	2.43	ND	1.21^c^
Mean	3.05^A^	ND	

T1: control, T2: pearl millet bran5%, T3: pearl millet bran10%, T4: pearl millet bran15%

Each value represents the mean of three replicates.

Means that have different lowercase letters (treatments) and uppercase letters (storage time) indicate significant differences at a significance level of *p* < 0.05.

### Color measurements of uncooked beef burgers

**Fig. 2:**
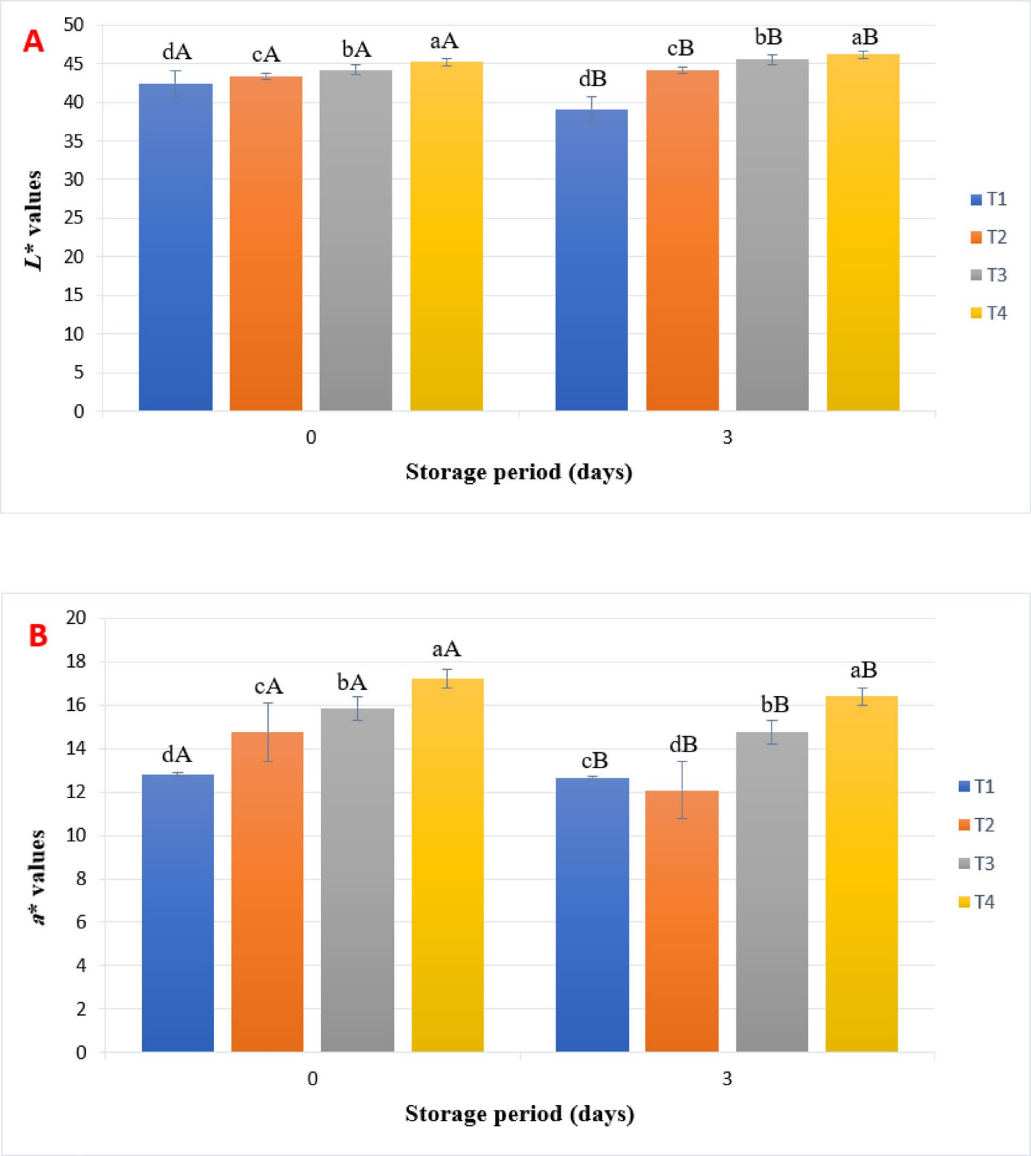

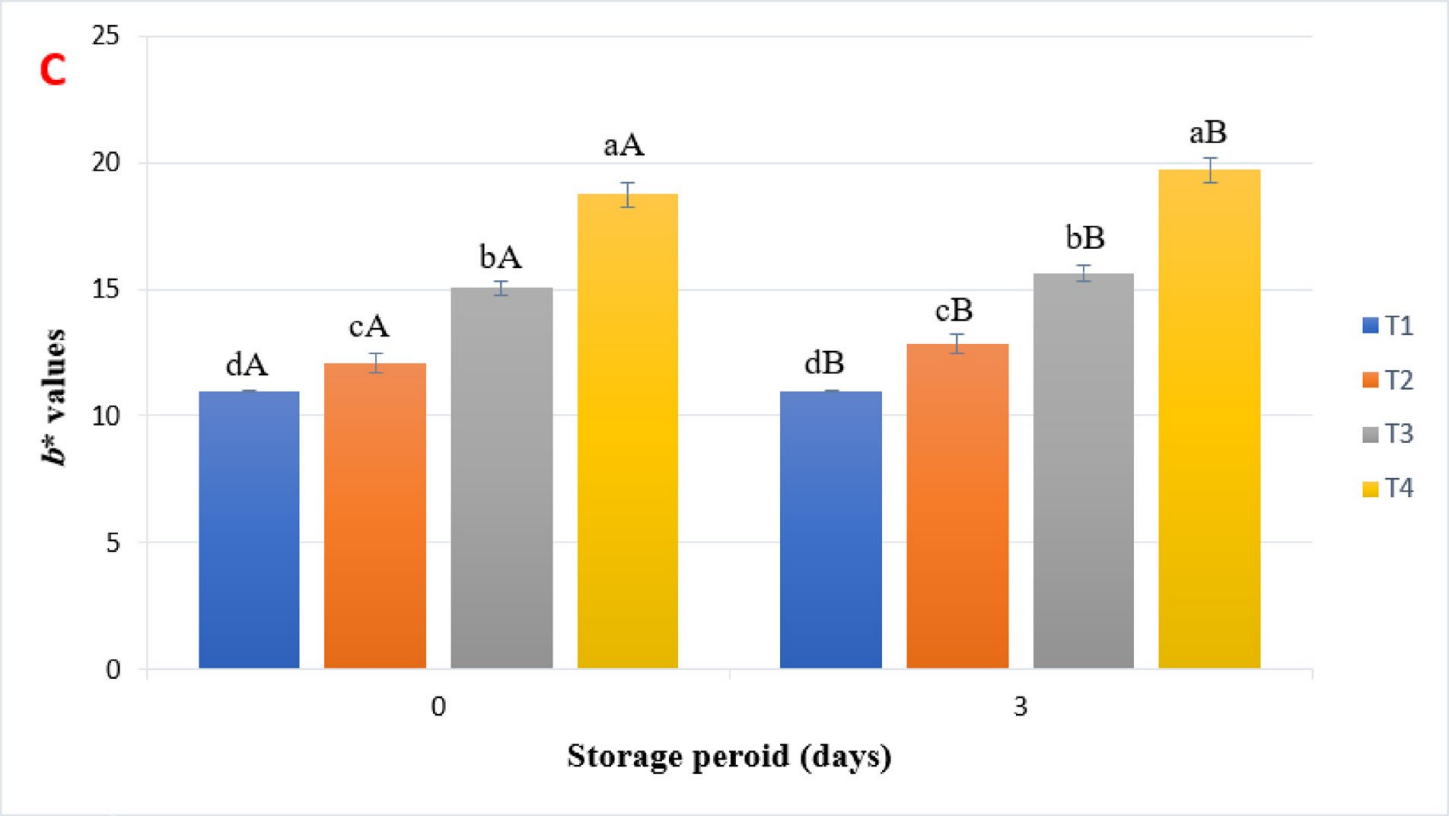
Changes in color parameters of uncooked beef burger samples during storage at 4 °C. T1: control, T2: pearl millet bran5%, T3: pearl millet bran10%, T4: pearl millet bran15%. Each value represents the mean of nine replicates ± SD. Means that have different lowercase letters (treatments) and uppercase letters (storage time) indicate significant differences at a significance level of *p* < 0.05.

The color of meat and meat products, influenced by the amount and form of myoglobin, can significantly impact consumers’ purchasing decisions^27^. Figure 2 demonstrates how adding pearl millet flour (PBF) affects the color parameters of beef burgers. Regarding lightness (L*), the addition of PBF increased the lightness of the beef burgers throughout the storage period, while the control samples (T1) experienced a decrease in lightness by the end of storage. The darkening observed in T1 may be attributed to myoglobin oxidation. The color of myoglobin can change dramatically depending on the oxidation state of iron^28^. In contrast, the antioxidant properties of millet bran may help maintain the lightness of the beef burgers. Among these antioxidants, phenolic compounds found in millet bran (Fig. 1) could play a key role in protecting the color of the burgers^8^. In terms of redness (a*), the results shown in Fig. 1 indicate that higher levels of millet bran addition resulted in increased redness values. At the beginning of storage, sample T4 exhibited the highest redness (a*) value of 17.22, while the control sample (T1) had the lowest value of 12.80. Notably, refrigeration did not affect the redness between samples. These findings are consistent with those reported by Huanga et al*.*^29^, indicating that the higher redness values in the treatments could arise from the product’s brownish hue, influenced by the greyish-brown color of pearl millet flour. Regarding yellowness (b*), the control sample (T1) had the lowest b* value of 10.96, whereas the sample containing 15% PBF (T4) had the highest b* value of 18.74. The b* values increased over the storage period, with the control sample (T1) rising to 10.99 and the 15% millet bran sample (T4) increasing to 19.70 (Fig. 2).

### Cooking characteristics and firmness of beef burgers

**Fig. 3:**
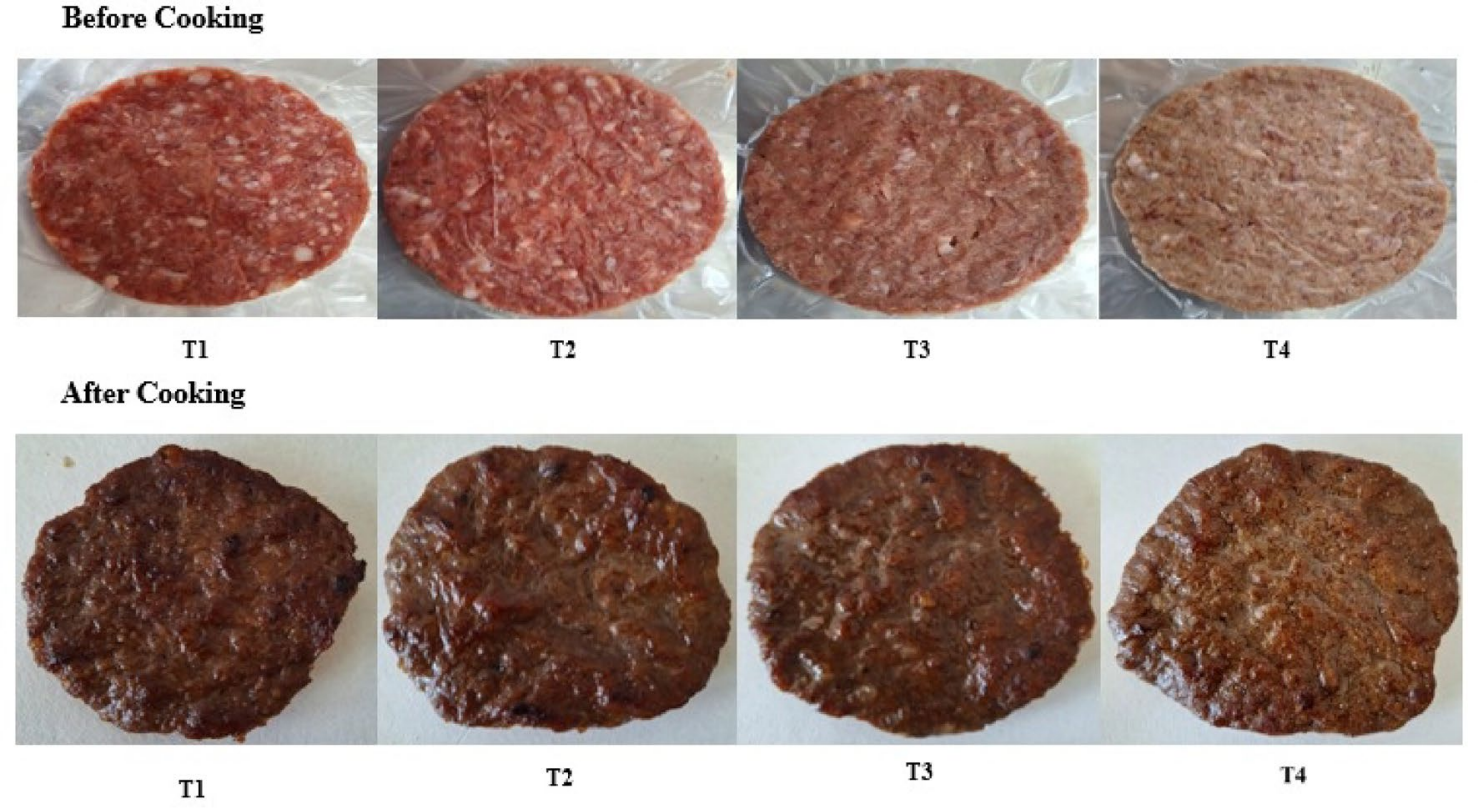
Shows the beef burger samples before and after cooking. T1: control, T2: pearl millet bran5%, T3: pearl millet bran10%, T4: pearl millet bran15%.

Table 4 and Fig. 3 shows the impact of adding millet bran on the characteristics of the cocked beef burgers. The addition of millet bran significantly affected the cooking loss of beef burger samples during cold storage (Table 4). Sample T4 exhibited the lowest cooking loss at 15.2%, while sample T1 had the highest cooking loss of 36.36%. Similar findings were reported by Ganguly^30^, who noted that adding pearl millet at different ratios (10–20%) increased the yield of chicken nuggets compared to the control sample. Furthermore, Aly^12^ found that incorporating pearl millet into burgers boosted their cooking yield. In all burger samples, cooking loss increased with extended cold storage periods. Cooking shrinkage, caused by the denaturation of proteins and the release of water and fat from the beef burger samples, is considered one of the most significant physical changes during the cooking process^31^. In this study, cooking loss increased with storage time for all samples (Table 4). The changes in diameter (shrinkage) of beef burger samples are presented in Table 4. Shrinkage was significantly reduced with a higher level of millet bran added. Sample T4 demonstrated the lowest shrinkage ratio at 13.29%, while the control sample (T1) exhibited the highest shrinkage ratio of 31.10%. The results also showed that shrinkage increased over storage time (Table 4). Bis-souza et al.^32^ and Aly^12^ reported that the shrinkage ratio of low-fat beef burgers fortified with dietary fiber decreased compared to the control sample.

**Table 4:** Cooking loss and diameter reduction of beef burger samples formulated with different concentrations of pearl millet bran during storage at 4 °C.

Treatments	Storage period (day)		Means
	0	3	
Cooking loss (%)			
T1	36.36 ± 0.41	32.76 ± 0.42	34.56^a^
T2	32.00 ± 0.65	24.05 ± 0.75	28.02^b^
T3	20.11 ± 1.31	18.62 ± 0.24	19.36^c^
T4	15.20 ± 0.10	10.40 ± 0.52	12.80^d^
Mean	25.92^A^	21.46^B^	
Diameter reduction (%)			
T1	31.10 ± 1.64	27.73 ± 0.65	29.41^a^
T2	25.56 ± 1.77	24.50 ± 0.64	25.03^b^
T3	18.84 ± 1.22	15.52 ± 1.39	17.18^c^
T4	13.29 ± 1.12	12.47 ± 0.72	12.88^d^
Mean	21.37^A^	20.88^B^	

T1: control, T2: pearl millet bran5%, T3: pearl millet bran10%, T4: pearl millet bran15%

Each value represents the mean of three replicates ± SD.

Means that have different lowercase letters (treatments) and uppercase letters (storage time) indicate significant differences at a significance level of *p* < 0.05.

**Fig. 4:**
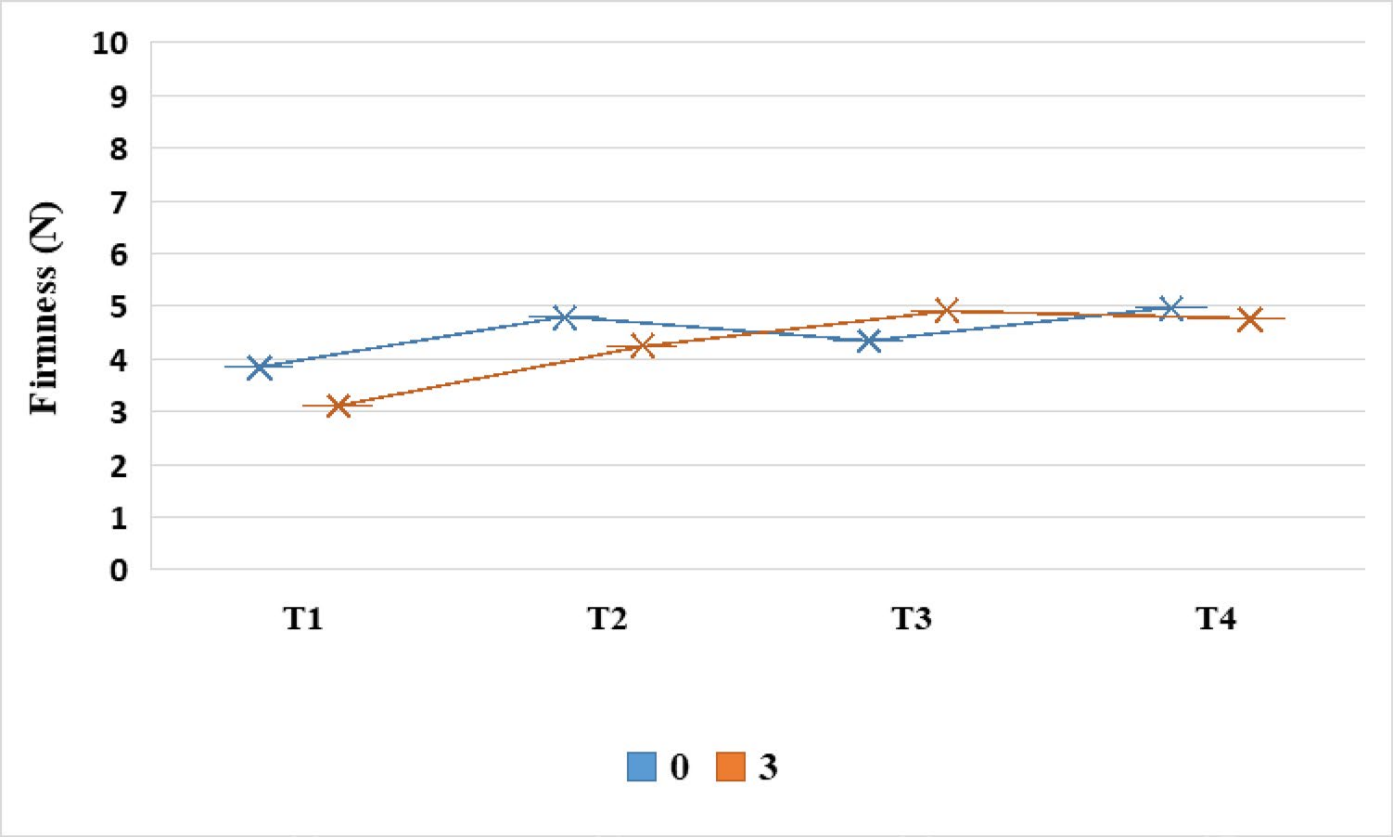
Firmness (N) of cooked beef burger samples formulated with different concentrations of pearl millet bran during storage at 4 °C. T1: control, T2: pearl millet bran5%, T3: pearl millet bran10%, T4: pearl millet bran15%. Each value represents the mean of three replicates ± SD.

The results presented in Fig. 4 illustrate the impact of pearl millet bran on the firmness of cooked beef burgers. The findings indicated that the firmness values rose as the levels of PMB increased. The control sample (T1) exhibited the lowest firmness value at 3.84 N, whereas the sample with 15% millet bran (T4) showed the highest firmness value at 4.96 N (Fig. 4). This increase in firmness might be attributed to the fiber content in pearl millet. However, Andrade et al.^5^ suggested that the variation in the properties of meatballs could be explained by the water-binding properties of certain nutrients found in plant ingredients, such as dietary fiber.

### Sensory evaluation of cooked beef burgers

Visual appearance, particularly color, significantly influences the organoleptic acceptance of meat and meat products^33^. The color, odor, taste, juiciness, tenderness, and overall acceptability of produced beef burgers are summarized in Table 5. The results indicate that adding millet bran improved the color scores compared to the control group (T1). This conclusion is further supported by the color parameters detailed in Fig. 1. As previously mentioned, antioxidants can enhance the color of myoglobin. To preserve the quality of meat products, myoglobin must be protected from oxidation before cooking^34^. The odor scores of the beef burgers are also presented in Table 5. Since the panelists were familiar with the natural odor of regular beef burgers, both T1 and T2 received the same odor score of 5.5 before storage. However, after several days of storage at 4 °C, the T2 samples exhibited the best odor score of 5.45. Similar results were observed regarding taste scores, as T1 and T2 had comparable scores during cold storage. Table 5 further illustrates the juiciness and tenderness of beef burgers. The results show that a low-level substitution of 5% millet bran led to increased juiciness and tenderness, likely due to the dietary fibers in millet bran. However, as the millet bran levels increased, juiciness and tenderness decreased. This reduction may be attributed to various factors, including the water-binding properties of dietary fiber and a decrease in fat and free moisture in samples with higher ratios of millet bran. These findings align with research by López-Vargas et al. (2014), which indicated that orange fiber’s capacity to retain water can make cooked sausages appear less juicy. Also, Aly et al.^12^ found that the juiciness of beef burgers fortified with pearl millet was noticeably lower than control samples, likely due to the reduced fat and moisture content. The changes in overall acceptability of beef burger samples during cold storage are presented in Table 5. All sensory attributes decreased across all samples as storage time progressed. According to the 1–7 point hedonic scale used in this study, the beef burgers from treatments T1, T2, and T3 were organoleptically acceptable during the three days of storage (Table 5). Notably, the T2 samples demonstrated superiority over the other samples. The high scores in color, odor, taste, juiciness, and tenderness contributed to the greater overall acceptability of the T2 beef burgers compared to the control and those with high millet bran levels in other treatments (Table 5). Similarly, Aly et al.^12^ reported that the higher levels of added pearl millet decreased the overall acceptability of beef burgers.

**Table 5:** Changes in sensory evaluation characteristics of beef burger samples formulated with different concentrations of pearl millet bran during storage at 4 °C.

Treatments	Storage period (day)		Mean
	0	3	
Color			
T1	5.87	5.37	5.62^ab^
T2	5.93	5.62	5.77^a^
T3	5.80	5.25	5.52^ab^
T4	4.62	4.80	4.71^b^
Mean	5.55^A^	5.26^B^	
Odor			
T1	5.50	5.17	5.33^c^
T2	5.50	5.45	5.47^b^
T3	4.87	4.85	4.86^a^
T4	4.75	4.12	4.43^b^
Mean	5.15^A^	4.89^B^	
Taste			
T1	5.52	5.40	5.46^b^
T2	5.62	5.52	5.57^a^
T3	4.62	4.62	4.62^c^
T4	3.87	3.75	3.81^d^
Mean	4.90^A^	4.82^B^	
Juiciness			
T1	6.00	5.62	5.81^b^
T2	6.50	5.37	5.93^a^
T3	5.56	4.87	5.21^c^
T4	4.75	5.00	4.87^d^
Mean	5.70^A^	5.21^B^	
Tenderness			
T1	5.25	5.50	5.37^b^
T2	5.87	5.62	5.74^a^
T3	5.43	5.37	5.35^b^
T4	4.87	5.25	5.06^c^
Mean	5.35^A^	5.43^B^	
General acceptability			
T1	5.55	5.47	5.51^b^
T2	5.91	5.50	5.70^a^
T3	5.26	5.00	5.13^c^
T4	5.57	4.60	4.58^d^
Mean	5.32^A^	5.14^B^	

T1: control, T2: pearl millet bran5%, T3: pearl millet bran10%, T4: pearl millet bran15%

Each value represents the mean ± SD.

Means that have different lowercase letters (treatments) and uppercase letters (storage time) indicate significant differences at a significance level of *p* < 0.05.

## Conclusion

The current study demonstrated that pearl millet bran can serve as a natural alternative to replace animal fat in beef burgers, enhancing their bioactivity as well as their chemical, physical, microbiological, and sensory qualities. Beef burgers fortified with a 5% addition of millet bran (T2) received the highest acceptance in organoleptic evaluations. Also, after three days of refrigerated storage, T2 samples consistently scored the highest in sensory attributes. These obtained results indicate that incorporation of pearl millet fiber present a good option to replace animal fat in beef burgers, improving cooking properties without causing any unfavorable effects in desirable textural and sensory quality characteristics. In addition, oxidation development occurred in meat products can be slowed down in processed meat products by adding pearl millet fibers. However, further research is needed to explore the effects of increased exploitation of pearl millet fiber in other processed meat products with extended shelf-life, in order to develop healthier options that may provide additional nutritional benefits while reducing the risks associated with diseases related to high fat content.

## Data Availability

All data are provided within the article.
